# Grapevine Potassium Nutrition and Fruit Quality in the Context of Climate Change

**DOI:** 10.3389/fpls.2020.00123

**Published:** 2020-02-26

**Authors:** Jérémy Villette, Teresa Cuéllar, Jean-Luc Verdeil, Serge Delrot, Isabelle Gaillard

**Affiliations:** ^1^ BPMP, Univ Montpellier, CNRS, INRAE, SupAgro, Montpellier, France; ^2^ CIRAD, UMR AGAP, Univ Montpellier, INRA, Montpellier SupAgro, Montpellier, France; ^3^ EGFV, Bordeaux Sciences Agro, INRAE, Université de Bordeaux, ISVV, Villenave d’Ornon, France

**Keywords:** potassium nutrition, potassium transport, fruit quality, grape berries, climate change

## Abstract

Potassium (K^+^) nutrition is of relevant interest for winegrowers because it influences grapevine growth, berry composition, as well as must and wine quality. Indeed, wine quality strongly depends on berry composition at harvest. However, K^+^ content of grape berries increased steadily over the last decades, in part due to climate change. Currently, the properties and qualities of many fruits are also impacted by environment. In grapevine, this disturbs berry properties resulting in unbalanced wines with poor organoleptic quality and low acidity. This requires a better understanding of the molecular basis of K^+^ accumulation and its control along grape berry development. This mini-review summarizes our current knowledge on K^+^ nutrition in relation with fruit quality in the context of a changing environment.

## Introduction

Plant growth and development rely on a balanced distribution of different mineral elements that are needed for various physiological processes. The beneficial effects of adding mineral elements to soils to improve plant growth has been known and used in agriculture for more than several thousand years. However, in the context of climate change, plants are facing increasing challenges to maintain balanced growth. High temperatures and soil water deficits associated with climate change affect agriculture and largely constrain plant productivity. Moreover, final product quality is also altered by excessive solar radiation, atmospheric CO_2_ levels, and rainfalls (Schultz, 2000; Korres et al., 2016). Grapevine (*Vitis vinifera*) is the most economically important fruit crop in the world. Wine grapes are particularly threatened by this phenomenon because its oenological potential is directly linked to its composition, in turn depending on pedoclimatic conditions (vintage effect). In this context, it is therefore important for the vine growers and wine makers to adapt many field practices including mineral nutrition.

Great wines are famous for their high standard and characteristic flavors that are distinguishable and reflect their environment (climate, soils, and grown varieties). In France, the southern regions have a typical Mediterranean climate with warm and long summers whereas central and northern regions undergo more humid climates. These climatic aspects have been properly taken into account in the past, to adapt various grapevine varieties and best suit their environmental conditions. But today the berry properties such as color, flavor, and aroma components are modified by the current climate environment, which results in unbalanced wines, with high alcoholic content and excessively low acidity (Jones et al., 2005). This is due to the regular increase of grape berry K^+^ and sugar contents that have been observed during the last decades.

Obviously, deficiencies in major minerals like potassium (K^+^), nitrogen (N), and phosphorus (P) strongly affect metabolism with subsequent impacts on plant growth, crop yield, nutritional value, and composition of grape berries (Amtmann and Armengaud, 2009; Fernandes et al., 2016). But with the effects of climate change, impact of mineral nutrition might change. This has been extensively studied for K^+^ nutrition because several reports mentioned that when K^+^ accumulation in grape berries at harvest is too high, berry acidity is too low (Conde et al., 2007; Walker and Blackmore, 2012).

Potassium is the major cation in plants. It can be present up to 10% of dry mass. It is a highly mobile osmolyte and a major component in the cation/anion balance. K^+^ is involved in neutralization of negative charges and organic acids and contributes to cellular turgor and elongation and mechanical movements such as stomata aperture or leaf movements (Maathuis, 2009; Sharma et al., 2013; Dreyer, 2014). Cytoplasmic concentration of K^+^ is maintained around 80 to 100 mM. Preserving this concentration range is important for many physiological processes as the control of electrical membrane potential, the maintenance of pH homeostasis in the cells, enzyme activations, and stabilization of protein synthesis. These different functions explain why K^+^ is so important for plants and is present in all tissues and subcellular compartments of cells. Indeed, it has been shown that plants accumulate large amounts of K^+^ in their vacuoles, surpassing purely nutritional requirements. K^+^ deficiency has negative impact on plant growth. K^+^ starvation reduces also the ability to use N and provokes chlorosis at the tip of older leaves in cereal crops, increasing crop’s susceptibility to diseases and affecting plant metabolism (Armengaud et al., 2009; Zörb et al., 2014; Coskun et al., 2017). Plant performance depends on K^+^ availability in soil and K^+^ uptake efficiency. To perform an optimal preservation of K^+^ homeostasis, a large group of K^+^ transporters and channels has been identified in plants. They are involved in K^+^ uptake by roots, ion translocation between organs and tissues, and storage in cellular vacuoles.

In grapevine, K^+^ plays an essential role in the initiation and control of massive fluxes that are necessary for berry growth during maturation (Cuéllar et al., 2013; Nieves-Cordones et al., 2019). In addition, grape acidity at harvest is a key factor to obtain wines of great quality. Grape acidity results from the ratio between free organic acids (i.e. malic and tartaric acids) and organic acids neutralized by K^+^. In the context of warmer climates, K^+^ ion accumulation increases during grape ripening, leading to an excessive neutralization of organic acids (Cuéllar et al., 2010; Cuéllar et al., 2013; Rogiers et al., 2017; Nieves-Cordones et al., 2019). Moreover, high temperature also affects the organic acid content of berries inducing the consumption of malic acid as a respiratory substrate (Famiani et al., 2016). This results in a low-acidic must context leading to the formation of insoluble K^+^ bitartrate during winemaking. Not only this amplifies the loss of acidity but this also gives rise to unstable wines with poor organoleptic properties. This is a major concern for grape production, although the molecular determinants that control berry acidity and K^+^ accumulation during ripening are still poorly known. Adaptation to climate changes is becoming a major challenge for grapevine. This mini-review focuses on K^+^ nutrition in grapevine in the context of our current environment and summarizes the knowledge available nowadays.

## Plants K^+^ Transport Systems and Physiological Functions

**Figure 1:**
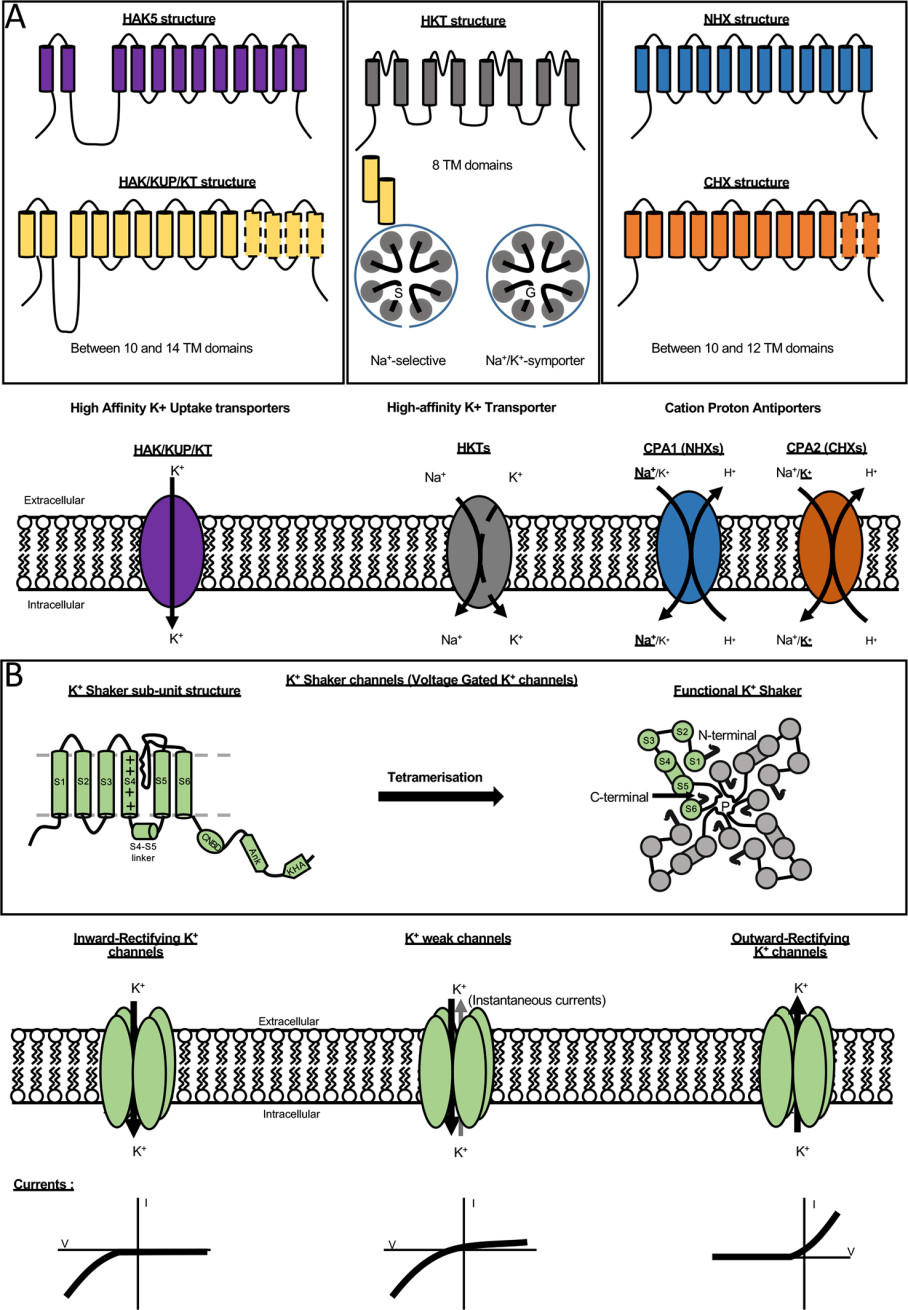
K^+^ transporter and channel families. **(A)** Structure and function of different transporter families. The family HAK/KUP/KT (left panel) is present in all plant genomes and contains 13 members in *A. thaliana* and 18 in vine distributed into five clades. Arabidopsis has just one member belonging to cluster I, AtHAK5, which has been extensively studied and its structure is shown in the top. The general structure of the HAK/KUP/KT transporters is conserved. The number of transmembrane segments (TMS) is ranged from 10 to14, with the most common being 11–12 TMS. The HKT (High-affinity K^+^ Transporter) transporters (middle panel) are distributed into two families depending of the presence of a glycine into the first pore loop proposed as a K^+^ selectivity determinant. All members of HKT family are composed of eight transmembrane domains. The last family of K^+^ transporters is the CPA family (right panel) for Cation Proton Antiporters. CPAs are composed of 10 to 12 transmembrane domains. This family is divided in two sub-families (CPA1 and CPA2). CPA1 mainly consists in NHX antiporters (Na^+^(K^+^)/H^+^ exchangers). CPA2 is mainly constituted by CHXs (cation/H^+^ exchangers). Information on the CPA family is still fragmentary. **(B)** Description of K^+^ Shaker channels. The structure of different members is strictly conserved with six transmembrane domains. The fourth transmembrane segment (S4) harbors positively charged amino acids and acts together with S1, S2, and S3 as voltage sensor. The pore domain is located between the TMS5 and TMS6 domains. The C-terminal extremity of Shaker sub-unit is composed of three distinct domains: a cyclic nucleotide-binding domain (CNBD), an ankyrin domain allowing protein-protein interactions and a KHA domain rich in hydrophobic and acidic amino acids. Functional Shaker channels are multimeric proteins formed by the assembly of four Shaker gene products (Dreyer et al., 2019). In plants, three types of K^+^ Shaker channel localized at the plasma membrane have been exhaustively characterized: the inwardly, weak inwardly or outwardly rectifying K^+^ channels which drive inward or outward K^+^ fluxes across the plasma membrane according to the membrane potential.

In the model plant *Arabidopsis thaliana*, a total of about 70 K^+^ channels and transporters, differing in transport affinity, energetic coupling, voltage sensitivity, or ionic selectivity, have been identified (Mäser et al., 2001; Lebaudy et al., 2008; Sharma et al., 2013; Véry et al., 2014). In grapevine, because excess of K^+^ levels in berries may have a negative impact on wine quality, molecular determinants of K^+^ transport are under investigation. Plant major multi-gene families encoding K^+^ permeable transport systems belong to one of the following five families (i) HAK-KUP-KT transporters, (ii) HKT transporters, (iii) cation-proton antiporters (CPA), (iv) Shaker-like K^+^ channels, and (v) two pore K (TPKs) channels. This review only focuses on the three transporters families and the K^+^ Shaker channels which are briefly presented below (**Figure 1**, Mäser et al., 2001; Sharma et al., 2013; Véry et al., 2014).

### K^+^-Selective Transporters HAK-KUP-KT

The HAK-KUP-KT family is usually selective for K^+^. Plant HAK-KUP-KT proteins possess 10 to 14 transmembrane domains with both N- and C-termini at the membrane intracellular side. These transporters are present in plants, fungi, bacteria, and even viruses but not in animals. They are crucial for organisms facing external solutions containing very low K^+^ concentrations (µM range) (Nieves-Cordones et al., 2010). In flowering plants, the HAK-KUP-KT family displays variable number of members: 13 genes have been identified in *A. thaliana* and 18 in grapevine which are classified among clades I-V (Nieves-Cordones et al., 2016). High-affinity K^+^ transport has been demonstrated for all members of the clade I sub-family to which belongs, e.g. HAK5 (high affinity K^+^ 5). HAK5, located at the plasma membrane, contributes to high affinity K^+^ uptake from soil and to root elongation (Ragel et al., 2019). HAK5 transporters are transcriptionally up-regulated under K^+^ starvation conditions in *A. thaliana*, but also in agronomic crop species like rice, barley, pepper, and tomato (Santa-María et al., 1997; Bañuelos et al., 2002; Martínez-Cordero et al., 2004; Nieves-Cordones et al., 2008). Moreover, HAK5 is also up-regulated in -K, -N, and -P starvation in tomato, whereas in *A. thaliana*, HAK5 is up-regulated only in -K^+^ and -N deficiencies (Rubio et al., 2014). This suggests different responses and regulation between plant species.

In *A. thaliana*, the 12 other members of this family are present in different tissues and are probably involved in many diverse physiological functions in plants (Ahn et al., 2004). Among them, AtKUP2, 4, 6, and 7 are involved in cell enlargement, K^+^ translocation, and long-distance K^+^ transport (Han et al., 2016; Santa-María et al., 2018). Some studies showed a large reduction of HAK-KUP-KT transcripts under high salt conditions and K^+^ deficiencies (Nieves-Cordones et al., 2010; Li et al., 2018). An interplay between sodium (Na^+^) and K^+^, at elevated concentrations of Na^+^, is described to have a negative effect on the expression of members of this family. In maize, it has recently been shown that some HAK transporters can transport Na^+^ instead of K^+^ (Zhang et al., 2019). These transporters belong to clade IV of the HAK-KUP-KT family. In grapevine, only one member was identified in this sub-group (Benito et al, 2012).

In grape, only two HAK-KUP-KT‐type K^+^ transporters have been studied so far. They are expressed most highly in the berry skin during the first phase of berry development (Davies et al., 2006) and therefore do not participate to the berry loading after veraison (the onset of ripening).

### Non-Selective Cation-Transporters HKT and CPA

Transporters of the HKT (high-affinity K^+^ transporter) and CPA families display varying permeabilities for K^+^ and Na^+^.

The HKT transporters can be divided into two sub-families; “Na^+^-selective transporters” or “Na/K^+^”-symporters. The Na^+^ selective transporter sub-family (sub-family I) is found in all higher plant species whereas the sub-family II which contains Na^+^/K^+^ symporters and K^+^-selective transporters, has been so far only identified in the monocotyledonous plants. The division into the two sub-families is associated with a molecular determinant of permeability to K^+^ that has been identified in the selectivity filter. The key amino acid of this determinant, located in the first pore loop, is a serine for all members of sub-family I which is replaced by glycine for the Na^+^/K^+^ symporters (sub-family II) (Mäser et al., 2002; Platten et al., 2006). In *A. thaliana*, a single HKT gene has been identified in the sub-family I, and is a strong actor of the tolerance to salinity in this species (Berthomieu et al., 2003). In grapevine, six HKTs have been identified, all belonging to the sub-family I. One of them, named VisHKT1:1, has been shown to be involved in the control of leaf Na^+^ exclusion and so in Na^+^ tolerance (Henderson et al., 2018). Water shortage induced by climate change leads to the use of water containing high levels of sodium chloride in some countries. The HKT1 K^+^ transporter may be a good candidate to avoid Na^+^ toxicity (Henderson et al., 2018). In the monocotyledonous crop species, several genes distributed into the two sub-families have been identified, e.g. rice (eight or nine genes depending on the cultivar) or wheat (nine genes) (Véry et al., 2014). The members belonging to the sub-family II are thought to play an important role in the regulation of K^+^ homeostasis at saline conditions in different species (Platten et al., 2006; Hamamoto et al., 2015; Cao et al., 2019).

The CPA family is divided in two sub-families. In *A. thaliana*, there are the NHX (Na^+^/H^+^ exchanger) antiporter sub-family, composed of eight members, and the CHX antiporter (cation/H^+^ exchanger) sub-family, including 33 members (Pardo et al., 2006). These two sub-families are composed of cation transporters present in the endomembranes as AtNHX5 and AtNHX6 (Dragwidge et al., 2019), at the vacuolar membrane as AtNHX1 or AtNHX2 (Bassil et al., 2011a), or at the plasma membrane like AtCHX17 (Chanroj et al., 2013). Some of these antiporters are involved in the regulation of K^+^ homeostasis, intravacuolar and cellular pH homeostasis, cell expansion, and salt stress tolerance (Chanroj et al., 2012). This family of antiporters is not well characterized, but some studies with double knock-out mutants showed disturbed phenotypes in *A. thaliana* (Apse et al., 1999; Bassil et al., 2011a; Bassil et al., 2011b). In grapevine genome, six genes belonging to NHX sub-family, distributed in two distinct groups: vacuolar (VvNHX1-5) and endosomal members (VvNHX6) (Ayadi et al., 2020) and eight genes belonging to CHX sub-family have been identified (Ma et al., 2015). One member of the NHX family (VvNHX1) which is expressed in the tonoplast of mesocarp cells, is involved in the accumulation of K^+^ in the vacuole and in the control of homeostasis of cytosolic K^+^ during grape ripening (Hanana et al., 2007).

### Voltage-Gated K^+^ Shaker Channels

Shaker channels are the best-characterized family related to K^+^ transport in plants. These channels dominate K^+^ fluxes in plants and are crucial to drive sustained fluxes across the plasma membranes. In *A. thaliana*, nine sub-units have been identified and characterized. Functional channels are tetrameric proteins arranged around a central pore (Dreyer et al., 2004; Sharma et al., 2013; Véry et al., 2014) and are composed by assembling of four Shaker subunits encoded either by the same gene (homomeric channel) or by different genes (heteromeric channel). Heterotetramerization is known to increase channel functional diversity (Jeanguenin et al., 2008; Sharma et al., 2013; Véry et al., 2014). Depending on their functional features, these channels drive inwardly or outwardly rectifying K^+^ fluxes according to the plasma membrane potential. Thereby, they mediate K^+^ translocation out of or into the cell (Sharma et al., 2013; Véry et al., 2014).

In addition to their functional properties, it is worth to note that the role of these channels *in planta* strongly depends on the tissue in which they are expressed. Indeed the same channel, expressed in both the phloem and stomata, is involved in the two different physiological processes taking place in these tissues. For example, one recent study focusing on the phloem highlights the importance of the outward Shaker channel GORK, which drives K^+^ efflux, in the membrane repolarization (Cuin et al., 2018) whereas this same channel, expressed in guard cells, is involved in stomatal closure (Hosy et al., 2003). In *A. thaliana*, the underlying mechanisms controlling stomatal movements have been extensively studied in relation with the K^+^ transport. Whereas GORK is the only outward K^+^ Shaker channel controlling stomatal closure, the inward-rectifying K^+^ currents involved in stomatal opening are driven by multiple inward Shaker channels named KAT1, KAT2, and AKT1 (Lebaudy et al., 2008). Otherwise, AKT1 is also expressed in the root where it is involved in K^+^ uptake from the soil and its activity is regulated by interacting with different CIPK/CBL complexes (Xu et al., 2006). In tomato and grapevine, orthologous K^+^ channels of AKT1, named LKT1 and VvK1.1, respectively, have been shown to be involved in the K^+^ uptake from the soil but also in pips K^+^ accumulation in grapevine (Hartje et al., 2000; Cuéllar et al., 2010).

Similarly to *A. thaliana*, the grapevine genome also contains nine Shaker genes coding for nine channel subunits with a number of members within each Shaker sub-family that is not strictly conserved between the two species. The VvK1.2 channel (an AKT1-like channel), is only expressed in the plasma membrane of the grape berry flesh cells and its unique function is to load K^+^ ions into these cells (Cuéllar et al., 2013). The VvK3.1 channel which, as its most related *A. thaliana* AKT2 channel, forms a weakly rectifying channel, giving rise to two current components with different gating modes. This unique property allows the AKT2-like channels to mediate inward as well as outward K^+^ currents. These channels are known to dominate phloem K^+^ conductance where they have a major role in phloem K^+^ loading and unloading (Marten et al., 1999; Lacombe et al., 2000; Ache et al., 2001; Hafke et al., 2007). In grapevine, VvK3.1 channel mediates K^+^ unloading in the berries and is also involved in the maintenance of transmembrane K^+^ gradients of phloem cells, which refers to the concept of K^+^ battery (Gajdanowicz et al., 2011; Dreyer et al., 2017; Nieves-Cordones et al., 2019). The K^+^ battery explains how an open AKT2-like channel can compensate for the reduced pH gradient present under energy limitation and can provide additional energy stored in the K^+^ gradient between the phloem cytosol and the berry apoplast for transmembrane transport processes (Dreyer et al., 2017). This plays a major role in driving sugar, amino acid, and water transport across plant cell membranes during phloem unloading and allows the phloem stream flux toward the berry may persist over a long period of time. Finally, four K^+^ outward Shaker subunits have been identified in grapevine whereas only two outward subunits exist in *A. thaliana.* Among them, the VvK5.1 Shaker exhibits an expression territory strongly enlarged in comparison with SKOR, its most related *A. thaliana* gene (Villette et al., 2019). As SKOR, this grapevine Shaker is also involved in K^+^ root to shoot translocation but its larger expression profile reveals new roles not described so far, e.g. the involvement of a K^+^ Shaker in the process of lateral root primordium development (Villette et al., 2019). In response to climate change, K^+^ transporters and particularly K^+^ Shaker channels identified in grapevine appear to be key molecular actors to sustain a suitable K^+^ uptake and distribution.

## Grapevine K^+^ Nutrition in the Context of Climate Change

The current climatic context with increasing temperatures and carbon dioxide levels, ozone depletion, and decrease in precipitation patterns is becoming a major challenge for agriculture (Korres et al., 2016). These adverse conditions influence productivity by affecting overall plant performance and disturbing plant phenology. Obviously, grapevine is affected by this climatic conditions, and doubly so, since wine production depends on both maintaining a good yield and grape composition at harvest.

**Figure 2:**
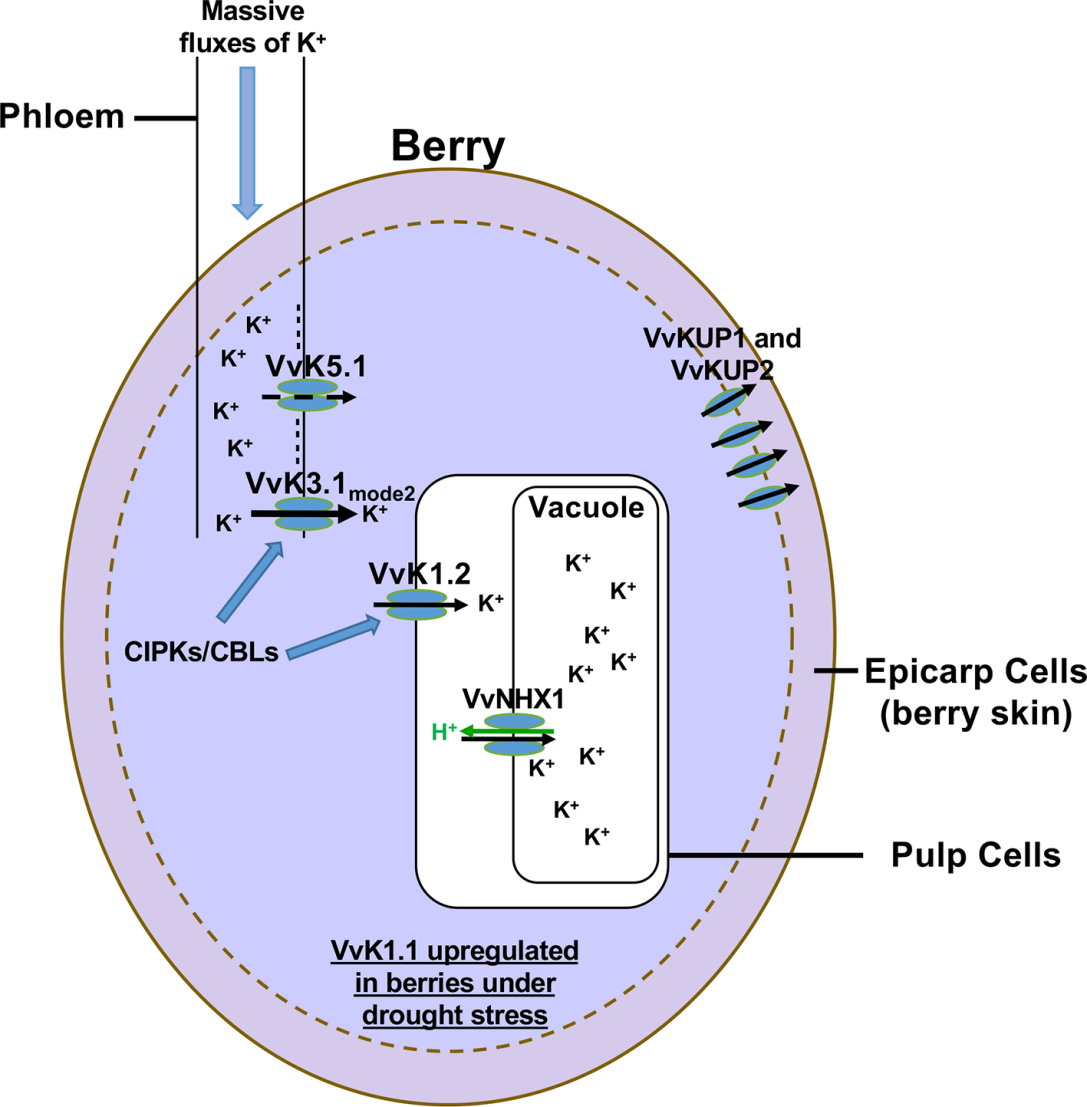
Map of K^+^ transport in grape berry. Seven K^+^ transport systems have been identified and characterized to be involved in K^+^ fluxes into or out of the berry cells. The first identified K^+^ transport systems belonging to the HAK/KUP-KT family are VvKUP1 and VvKUP2. These transporters are involved in K^+^ transport into the berry skin only during the first phase of berry development. In contrast, three K^+^ Shaker channels and one antiporter belonging to the CPA family have been characterized to be involved in K^+^ berry loading during its ripening. Recently, two other Shaker channels have been characterized in phloem cells. VvK5.1, which is a typical outwardly rectifying K^+^ channel, is involved in the repolarization of the plasma membrane of phloem cells (Villette et al., 2019). The second one, VvK3.1, is a weakly rectifying K^+^ Shaker channel that can switch between two gating modes driving either inwardly rectifying or instantaneous currents. The latter mode allows to drive K^+^ influx or efflux through phloem cell membranes according to membrane potentials and K^+^ gradients. At the unloading site, the K^+^ gradient is in favor of K^+^ efflux (100 mM in the cytosol of phloem cells and 1 mM in the apoplast). The VvK3.1 channel is the main molecular actor involved in K^+^ unloading into berries, thanks to massive K^+^ efflux into the apoplast (Nieves-Cordones et al., 2019). Then, apoplastic K^+^ is directly recovered by the inwardly rectifying K^+^ channel VvK1.2 expressed in pulp cell plasma membranes. The CPA transporter, VvNHX1, expressed in the tonoplast of pulp cells, is involved in K^+^ storage in the vacuole during berry ripening (Hanana et al., 2007). It is important to note that some members of the CIPK/CBL families increase the functional activity of VvK3.1 and VvK1.2. Finally, another inwardly rectifying K^+^ channel, named VvK1.1, involved in K^+^ uptake from the soil in roots, is up-regulated in berries in drought stress conditions (Cuéllar et al, 2010).

Among the climate parameters that affect the most the berry content at harvest, temperature and water availability play a prominent role. High temperature affects the phenology of grape berry development and ripening, resulting in a shift of picking dates toward earlier periods depending on the region, the variety, and the wine type. Heat waves directly increase sugar import (Mori et al., 2007; Parker et al., 2011) and K^+^ accumulation (Mpelasoka et al., 2003; Cuéllar et al., 2010; Kodur, 2011; Cuéllar et al., 2013; Rogiers et al., 2017; Nieves-Cordones et al., 2019). Phloem transport and berry metabolism also increase at high temperature. From the onset of the ripening (veraison), berry loading is ensured from the phloem by a directional flow of water, sugar, and nutrients driven by massive K^+^ fluxes (Nieves-Cordones et al., 2019). In addition, it is also known that vineyards grown in warmer areas give wines with low acidity. This is due to an excessive accumulation of K^+^ leading to an increase of the electrical neutralization of organic acids, which disturbs the control of the pH and of the acid-base balance of the flesh cells (Kodur, 2011). High temperatures also decrease the atmospheric water potential and favor plant transpiration and soil dehydration, which in turn favor water stress. Upon drought stress, it is worth to note that the shaker channels VvK1.1, VvK1.2, and VvK3.1, whose expression located in berry phloem vasculature or in berry flesh cells, are strongly up-regulated (Cuéllar et al., 2010; Cuéllar et al., 2013; Nieves-Cordones et al., 2019).These Shaker channels could thus play a role in K^+^ loading into berries during drought stress. We (and others) have invested in the identification of K^+^ transport systems and molecular regulators involved in grape berry K^+^ loading. Current understanding of these processes is summarized in **Figure 2**.

Plant responses to environmental stresses under a K^+^-limiting scenario are poorly understood. Field studies have observed that K^+^ as an osmolyte, can enhance osmotic adjustment or osmoprotection by maintenance of leaf turgor. This is directly linked to the improved capacity to retain water (Babita et al., 2010; Levi et al., 2011). Moreover, Rivas-Ubach et al. (2012) showed that in leaves of *Erica multiflora* (Heather) K^+^ concentrations increase under warm and drought conditions, in agreement with the role of K^+^ in osmotic protection. However, in *Olea europea* (olive) the stomatal conductance is disturbed when plants grow under moderate K^+^ deficiency in water stress conditions (Arquero et al., 2006). This behavior suggests that plants are impaired in their ability to grow during drought conditions when it is not possible to take up K^+^. This could explain why in grapevine, the berry K^+^ concentration during ripening enhances when high temperatures are combined with water stress. This higher K^+^ accumulation should contribute to a better osmotic adjustment in grape berry cells. In the context of climate change, many fruits present similar symptoms and suffer of detrimental effects that greatly affect their features and qualities. Under high temperature exposure, during maturing, apples become less acid with a decrease of fruit firmness and watercore (Sugiura et al., 2013). In sweet orange (*Citrus sinensis*) and clementine (*Citrus clementin*a) sugar content increases and fruit acidity decreases (Albrigo and Galán Saúco, 2004; Cercós et al., 2006). During flowering, high temperatures can have a deleterious impact on olive production (Gabaldón-Leal et al., 2017). Altogether, understanding the underlying mechanisms to face to the climate change is now an important challenge for fruit production.

## Author Contributions

All authors listed have made a substantial and intellectual contribution to the work, and approved it for publication.

## Funding

This work was supported by SweetKaliGrape ANR (ANR-33 14-CE20-0002-02). JV was the recipient of a PhD fellowship from the Institut National Recherche Agronomique and from Agropolis fondation in the context of APLIM (Advanced Plant Life Imaging) project (contract 1504-005).

## Conflict of Interest

The authors declare that the research was conducted in the absence of any commercial or financial relationships that could be construed as a potential conflict of interest.
